# *STAT4* Gene Variant *rs7574865* Is Associated with Rheumatoid Arthritis Activity and Anti-CCP Levels in the Western but Not in the Southern Population of Mexico

**DOI:** 10.3390/genes15020241

**Published:** 2024-02-14

**Authors:** Karla Mayela Bravo-Villagra, José Francisco Muñoz-Valle, Christian Johana Baños-Hernández, Sergio Cerpa-Cruz, José Eduardo Navarro-Zarza, Isela Parra-Rojas, José Alonso Aguilar-Velázquez, Samuel García-Arellano, Andres López-Quintero

**Affiliations:** 1 Instituto de Nutrigenética y Nutrigenómica Traslacional, Centro Universitario de Ciencias de la Salud (CUCS), Universidad de Guadalajara (UdeG), Guadalajara 44340, Mexico; karla.bravo2318@alumnos.udg.mx; 2 Doctorado en Genética Humana, Centro Universitario de Ciencias de la Salud (CUCS), Universidad de Guadalajara (UdeG), Guadalajara 44340, Mexico; josealonso.aguilarvelazquez@academicos.udg.mx; 3 Instituto de Investigación en Ciencias Biomédicas, Centro Universitario de Ciencias de la Salud (CUCS), Universidad de Guadalajara (UdeG), Guadalajara 44340, Mexico; drjosefranciscomv@cucs.udg.mx (J.F.M.-V.); johana.banos@academicos.udg.mx (C.J.B.-H.); samuel.garcia4566@academicos.udg.mx (S.G.-A.); 4 Antiguo Hospital Civil de Guadalajara “Fray Antonio Alcalde”, Guadalajara 44200, Mexico; scerpa@hcg.gob.mx; 5 Hospital General “Raymundo Abarca Alarcón”, Chilpancingo de Bravo 39016, Mexico; eduardo@navarrozarza.com.mx; 6 Facultad de Ciencias Químico Biológicas, Universidad Autónoma de Guerrero, Chilpancingo de Bravo 39086, Mexico; iprojas@yahoo.com

**Keywords:** rheumatoid arthritis, DAS28, RF, CRP, anti-CCP antibody, *STAT4*

## Abstract

Rheumatoid Arthritis (RA) is a multifactorial autoimmune disease. Currently, several genes play an important role in the development of the disease. The objective was to evaluate the association of the *STAT4 rs7574865* and *rs897200* gene variants with RA susceptibility, DAS28, RF, and anti-CCP in Western and Southern Mexico populations. Genotyping was performed on 476 samples (cases = 240; controls = 236) using the Taqman^®^ system and qPCR probes. Disease activity was assessed using DAS28 and HAQ DI. CRP, ESR, RF, and anti-CCP were determined for clinical assessment. Our study showed there is a statistically significant association with susceptibility to RA for the *rs7574865* variant in the Western population for the GT and TT genotypes. The same genotypes also showed a moderate-to-high activity according to DAS28 and positive anti-CCP compared to the control group. This association was not found in the Southern population. This work confirms the association of the *rs7574865* variant with RA, as well as a moderate-to-high activity and positive anti-CCP in the Western population but not in the Southern population. No association of the *rs897200* variant was found in any of the studied populations.

## 1. Introduction

Rheumatoid Arthritis (RA) is an autoimmune and multifactorial inflammatory disease, characterized by synovial inflammation, inflamed joints, and cartilage and bone structure deformation [1,2]. In 2010, The American College of Rheumatology (ACR) and the European League Against Rheumatism (EULAR) proposed criteria for RA diagnosis. These guidelines aim to identify RA and require at least six points to diagnose patients [3] considering the following categories: 1. Amount and location of joints affected. 2. Serological abnormalities (RF and anti-CCP). 3. Elevated inflammatory markers (CRP and ESR). 4. Symptom length [4].

The development of RA is multifactorial, of which the following are some of the most important factors: (1) strongly associated with genetic factors such as genetic variants; (2) environmental factors, such as occupational exposure, smoking and bacterial and viral infections; and (3) intrinsic factors, such as age, gender, and ethnicity [3]. The prevalence of RA varies by country since environmental factors can induce its development. However, it is estimated that, on average, RA affects 1% of the world population [5]. In Mexico, a prevalence of 0.7 to 2.8% of patients was reported suffering from RA [6]. Geographical location and lifestyle are important factors that place Mexico as one of the countries with a high percentage of RA patients [6]. 

Currently, it is known through genome-wide association studies (*GWASs*) that there are more than 150 susceptibility *loci* for RA, such as the *HLA-DRB1*, *STAT4*, *PTPN22*, *PADI4*, and *TRAF1* genes [7,8,9]. The main associated *loci* with RA can be divided into HLA and non-HLA. The *HLA-DRB1* alleles represent the strongest genetic link to RA and are likely responsible for at least 30% of the overall genetic contribution to the development of this disease. It has been described that *HLA-DRB1* is associated with the risk of developing RA, with severity, and with patient mortality, especially in those with positive antibodies such as rheumatoid factor (RF) and anti-cyclic citrullinated peptide antibodies (anti-CCP). Other associations related to HLA alleles, tobacco exposure, and response to biological treatments have been investigated. In patients with RA, resistance to biological treatment may develop through the generation of antidrug antibodies shortly after starting the treatment. However, the presence of these antibodies may decrease if administered concurrently with other immunosuppressive treatments, such as methotrexate [7,10,11,12,13,14]. Additional genes with smaller effects have been identified in the context of RA; a large number of these have a role in immune system regulation and inflammatory responses. Previous research has identified that certain non-HLA genes, such as *PTPN22*, *PADI4*, *TRAF1*, and *STAT4*, have a greater relationship with RA. These genes collectively contribute to an increased genetic predisposition, which suggests a complex interplay of genetic factors in the development of the disease [2,8,15]. The *PTPN22* gene is the second-most relevant susceptibility *locus* associated with RA; the presence of the *C1858T* variant in this gene results in a change from arginine to tryptophan, causing a loss of function which leads to the expansion of T cells and predisposes individuals to the development of autoimmunity [16,17]. In the case of *PADI4*, variants in this gene can convert arginine to citrulline that generates citrullinated proteins, which may contribute to the loss of immune tolerance and the synthesis of anti-CCP antibodies. The detection of anti-CCP antibodies is crucial for accurate diagnosis and prognosis in RA cases [18,19]. The *TRAF1* gene is implicated in RA as well, where some variants can create binding sites for the EP300 protein that regulates transcription through chromatin remodeling, suggesting a role in the dysregulation of gene expression in RA [20,21]. Finally, the *STAT4* gene participates in the differentiation and proliferation of Th1 and Th17 cells, playing a crucial role in the development of autoimmune diseases, including RA. Understanding the impact of these genes with smaller effects contributes to a more comprehensive understanding of the complex genetic factors underlying RA [22,23]. The *STAT4* gene, located in the *2q32.2* cytogenetic band, consists of 24 exons and codes for the transcription factor called “signal transducer and activator of transcription 4” (*STAT4*) [15,24,25]. This gene significantly influences the pathogenesis of RA by activating the JAK/STAT signaling pathway. This pathway is activated by signals induced by several cytokines, including IL-12, IL-23, and IFN-γ. The activation of this pathway contributes to the differentiation and proliferation of Th1 and Th17 cells, which are crucial in the development of chronic inflammatory and autoimmune diseases [26,27]. 

Within the *STAT4* gene, two genetic variants have been described that are involved in autoimmune diseases. The *rs7574865* variant is associated with various autoimmune diseases, with systemic lupus erythematosus (SLE) and RA being among the most common. It is located in intron 3 of the *STAT4* gene and is a single nucleotide variant with a T > G change, with a minor allele frequency of 0.26. Although the functional implication of this variant is still not clear, it is hypothesized that the presence of the risk allele increases the expression of the *STAT4* gene, leading to a higher phosphorylation of STAT4 (p-STAT4) and IFN-γ production in T cells [28,29,30]. On the other hand, the *rs897200* variant is associated with Behcet’s syndrome (BD). It is located 1846 bp upstream of the *STAT4* gene, a single nucleotide variant with a T > C change and a minor allele frequency of 0.50. This variant, in the presence of the risk allele, may confer an increased risk by influencing the expression of *STAT4*. Studies have suggested that the risk conferred by the less frequent allele is associated with the overexpression of *STAT4* and subsequent transcription and protein expression of IL-17 since *STAT4* induces the differentiation of T cells into the Th1 or Th17 phenotype [31,32]. 

Interleukin activation during the inflammatory process in RA causes a cascade effect and activates a signaling pathway known as JAK/STAT [26,33], which uses cytokines as an immune and inflammatory response. Each JAK protein is specific for cytokine receptors [27]. STAT proteins are initially inactive cytoplasmic proteins, but once cytokines bind to their specific receptors, activation of the JAK/STAT pathway occurs, leading to the phosphorylation of p-STAT dimers. This forms homodimers or heterodimers of STAT proteins that can then translocate to the nucleus and act as transcription factors in specific genes. Therefore, certain cytokines play an important role in the pathogenesis of RA [33]. Thus, the objective of this study was to establish if there is an association between two genetic variants *rs7574865* and *rs897200* of the *STAT4* gene with the susceptibility to develop RA through genetic models and clinical variables in the Mexican population. 

## 2. Materials and Methods

### 2.1. Study Participants 

This study included 476 Mexican participants from the Western (Jalisco) and Southern (Guerrero) states; 240 had RA, of which 120 were from the Western and 120 were from the Southern states. A control group of 236 healthy people was included, with 120 from the Western and 116 from the Southern states. RA patients were recruited from the Rheumatology Department of Hospital Civil “Fray Antonio Alcalde”, Guadalajara, Jalisco, and from the Rheumatology department at Hospital General of Chilpancingo “Dr. Raymundo Abarca Alarcón”, Chilpancingo, Guerrero. The following variables of each patient were considered: diagnosis according to ACR/EULAR 2010 criteria [19]. The data collected included demographics, clinical assessment such as time of disease evolution, severity and activity evaluated by the disease activity score (DAS28) (calculated considering the inflamed and painful joints), EVA and erythrocyte sedimentary volume (ESR) applied by the rheumatologist, analogous patient pain scale (EVA), health assessment questionnaire (HAQ), RA treatment and clinical variables for diagnosis such as rheumatoid factor (RF), anti-cyclic citrullinated peptide antibody (anti-CCP), c-reactive protein (CRP), and erythrocyte sedimentary volume (ESR). All the participants signed an informed consent and did not have overlapping rheumatic diseases, such as fibromyalgia, systemic lupus erythematosus, Sjögren’s syndrome, systemic sclerosis, ankylosing spondylitis, psoriatic arthritis, Behçet’s syndrome, or gout. The project was previously approved by the bioethics committee of the Centro Universitario de Ciencias de la Salud, Universidad de Guadalajara. 

### 2.2. Autoantibodies and Laboratory Assessment

The anti-CCP levels were measured using enzyme-linked immunosorbent assay (ELISA) (REF FCCP600; Axis-Shield Diagnostics Limited, The Technology Park, Dundee, D UK), and serum values >5 U/mL were used as a stringent criterion for positive anti-CCP. RF (IU/mL) and CRP (mg/L) were quantified using a turbidimetric assay (COD31922 and COD31921; BioSystems, respectively; Barcelona, Spain). The assay COD31922 is made of latex particles coated with human gamma-globulin, and serum values above 20 IU/mL were considered positive. Erythrocyte sedimentary volume (ESR) was determined using the Wintrobe method (mm/h) [34]. All determinations were performed at the Instituto de Investigación en Ciencias Biomédicas, Universidad de Guadalajara. 

### 2.3. Genotyping of STAT4 Variants (rs7574865 and rs897200)

A total of 476 DNA samples were genotyped using allelic discrimination by pre-designed TaqMan^®^ probes for *rs7574865* G/T (part number C_29882391_10; Applied Biosystems, Foster City, CA, USA) and *rs897200* C/T variants (part number C_7476952_10; Applied Biosystems, Foster City, CA, USA). The genotype of each sample was obtained automatically by measuring its allele-specific fluorescence using real-time PCR (LightCycler^®^; Roche, Barcelona, Spain).

### 2.4. Statistics

Genotypic and allelic frequencies of *rs7574865* and *rs897200* variants were determined by direct counting and the comparison of frequencies was carried out using the Chi-square test. The Hardy–Weinberg equilibrium was calculated using the Arlequin program v. 3.5.2.2. The distribution of all continuous variables was examined using the Kolmogorov–Smirnov test. The comparison of means for two independent samples was performed using Student’s *t*-test. The comparison of medians was performed using the Mann–Whitney–U test. Differences in genotypes, allelic frequencies, HAQ, EVA-PAC, lifestyle, and familial hereditary history were compared by using the Chi-square test through IBM SPSS and Graph Pad Prism 8. The contribution of genetic variants to the development of RA was evaluated through binary logistic regression considering a 95% Confidence interval (95% CI), and a *p*-value < 0.05 was considered statistically significant. 

## 3. Results

Demographic and clinical evaluation variables, such as age, gender, PCR, RF, and anti-CCP, were compared between cases and controls in the Western and Southern populations. Cases exhibited higher values for RF and anti-CCP compared to the control group for both populations, with a significant difference (<0.001). Additionally, a comparison was made between cases in both populations regarding variables such as family history, disease duration, DAS28, HAQ, VAS, ESR, and type of treatment, with significant values. These results are presented in Table 1. 

Table 2 displays the association results of genotypes for two variants, *rs7574865* and *rs897200*, in relation to DAS28, RF, and anti-CCP in both Western and Southern populations. Notably, no significant values were observed in the Southern population. In the Western population, significant results were obtained when dividing the values into two categories: remission or low (<3.2) and moderate to high (>3.2) for DAS28. The GT genotype (OR = 2.424, 95% CI 1.268–4.635) and TT genotype (OR = 3.967, 95% CI 1.597–9.854) of the rs7574865 variant showed significance in patients with moderate-to-high disease activity. Furthermore, when assessing anti-CCP, it was divided into negative (<5 IU/mL) and positive (>5 IU/mL) categories, and significant values were observed in the GT genotype (OR = 2.956, 95% CI 1.489–5.869) and TT genotype (OR = 3.024, 95% CI 1.137–8.046) for patients with positive anti-CCP in relation to the *rs7574865* variant.

The construction of haplotypes was performed, and the linkage disequilibrium for the Western and Southern populations was assessed, where no linkage disequilibrium was found for either of the two studied populations (D’ = 0.052 and 0.034, respectively).

**Figure 1:**
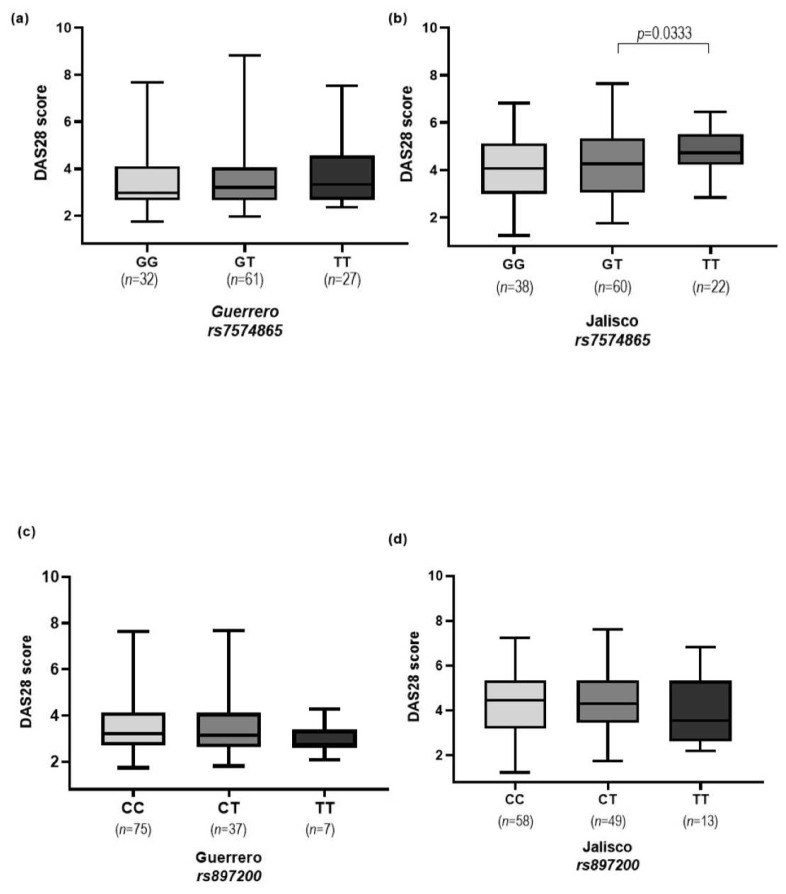
Distribution of *STAT4* gene variants in RA. (**a**,**b**) DAS28 score according to *rs7574865* genotypes in both populations (Western and Southern Mexico); a higher disease activity was found in TT carriers compared to GT carriers in the Jalisco population (*p* = 0.033) for *rs7574865* variant. (**c**,**d**) DAS28 score according to *rs897200* genotypes in both populations with no significant differences.

The relationship between the disease activity, measured by the DAS28 index, and the *STAT4* gene variants (*rs7574865* and *rs897200*) is presented in Figure 1. In the Western population, the GT and TT genotypes of the *rs7574865* variant showed significant values (*p* = 0.033) in terms of disease activity measured by the DAS28 index.

In the Southern population, no significant association was evident with the risk allele for either of the two variants. In the Western population, on the other hand, the *rs897200* variant did not show a significant relationship. However, concerning the *rs7574865* variant in the Western population, both the GT and TT genotypes exhibited a statistically significant association with RA as a risk factor when compared to the control group (OR = 2.198, 95% CI = 1.253–3.857, *p* = 0.005; OR = 3.243, 95% CI = 1.412–7.450, *p* = 0.004, respectively). Furthermore, the analysis of the dominant model (GT + TT vs. GG) indicated that the T allele is associated with susceptibility to RA (OR = 2.403, 95% CI = 1.412–4.090, *p* = 0.001). In the allele comparison, it was found that the T allele is also associated with susceptibility to the development of RA in the Western population (OR = 1.915, 95% CI = 1.308–2.806, *p* = 0.007). These results are presented in Table 3.

## 4. Discussion

The worldwide prevalence of RA is estimated to be 1% [35]. Prevalences of up to 6% can be found in Native American populations [36]. However, in Mexico, the prevalence varies between 0.7% and 2.8% [6]. Several factors contribute to the development of RA, including lifestyle, occupational activities, variations in access to healthcare systems, disparities in the rate of detection and diagnosis, as well as genetic factors. Heritability is approximately 40% to 60% in patients with RA who test positive for anti-CCP [35,36]. Additionally, genetic biomarkers can be used to determine the prognosis of the disease. Association studies have been conducted on various genetic variants of candidate genes, such as *PTPN22*, *HLA-DRB1*, *TNFAIP3*, *TRAFI*, and *STAT4* in different ethnic origins [37,38,39] 

Currently, there is a growing understanding of the genetic factors linked to the susceptibility and severity of RA. One of the factors involved in the pathophysiology of RA is the gene that encodes the transcription factor STAT4. This transcription factor is crucial in activating the JAK/STAT signaling pathway. This pathway can be activated by a range of stimuli, including hormonal influences, stressful circumstances, viral infections, growth factors, and neurotransmitters [40,41]. 

The cytokines that participate in the JAK/STAT pathway are implicated in many pain mechanisms, including those involving IL-6 and IL-1, which have been shown to impact cognitive function negatively. Moreover, the association between IFN-Ɣ and IL-12 has been established in the context of pain initiation or perpetuation. Therefore, it is imperative to investigate innovative targeted therapies that can efficiently suppress cytokines in the JAK/STAT pathway, which have a pivotal role in the pathogenesis of RA. The alleviation of pain symptoms can be achieved by limiting the activity of particular cytokines as evidenced by improvements in pain scores measured using the visual analog scale (VAS). New biological treatments would be particularly valuable for patients who do not respond positively to traditional DMARDs. This approach holds promise in improving the overall management of RA by targeting specific pathways involved in the disease pathogenesis. By focusing on cytokine inhibition, we can potentially reduce inflammation, slow down joint damage, and enhance the overall quality of life for RA patients. However, further research and clinical trials are needed to validate the efficacy and safety of these targeted treatments and to optimize their use in clinical practice [33,42,43,44].

The cause of early-onset RA cannot be defined, since it can be triggered by environmental factors such as exposure to tobacco smoke, mining and rock drilling, a low intake of vitamin D and antioxidants, red meat consumption, a high intake of sugar and salt, a lack of preventive access to healthcare, and a misdiagnosis of the disease [40,45,46]. 

The Mexican population is composed mainly of indigenous and Mestizo (admixed) populations, where the latter represent around 94% of the country’s total population. The Mexican–Mestizo population is the result of ~500 years of genetic admixture of Europeans (mostly Spaniards), Native American individuals, and, to a lesser extent, Africans. This extensive admixture process results in a distinct pattern of ancestry across the country, where Northern populations exhibit a higher European ancestry that gradually diminishes towards the South; contrarily, the Native American ancestry is higher in the South and decreases in the North, while the African ancestry is observed lower and heterogeneous [47]. Moreover, the ancestry of the current Mexican–Mestizo populations recapitulates the Native American substructure and affects some biomedical traits, which may explain at least partially the differences observed even in different Mexican populations [48]. The populations studied here have shown significant variations in their ancestral composition, where the Western population displays some of the higher European ancestry of the country [European (60–64%), followed by Amerindian (25–21%) and African (15%)], while the Southern population has a higher Native American ancestry and one of the higher African ancestries observed around the country [Amerindian ancestry predominates (48%), followed by European (38%), Asian (10%), and African (4%)] [49,50,51,52].

We carried out research in two distinct Mexican communities, one located in the South (Guerrero) and the other in the West (Jalisco). Both populations in this study had an average BMI >25, indicating that they were overweight. According to several authors, women with a BMI >30 may have a higher risk of developing RA, while for men, it appears to be a protective factor [40,41]. Most of the participants in this study were women, and the average age of onset they presented was 38 years and 42 years for the Western and Southern populations, respectively. Globally, the age of disease onset is 46 years [53], whereas in different parts of Mexico, it varies between 55 and 65 years [6]. Age can be considered a risk factor in women as it is associated with the onset of menopause, where there is a decrease in estrogen, an important hormone in the immune system [6,41,45,54]. 

In addition, a comparative analysis of wood smoke exposure during cooking was conducted, but no statistically significant differences were observed. Furthermore, it is worth noting that 27% of the individuals diagnosed with RA in the Western population were smokers, whereas just 5% of the participants in the Southern region smoked (*p* = 0.001). Several studies have linked heavy and prolonged tobacco smoke exposure to a 2.54-fold higher risk of developing RA. This is primarily attributed to the presence of Cadmium in tobacco leaves, which catalyzes reactive oxygen species or replaces essential cofactors like Zn, Cu, and Mn in antioxidant enzymes. This activation triggers an inflammatory process through cytokines such as IL-8 and TNF-α, leading to the infiltration of neutrophils and macrophages into the synovial membrane and an increased production of anti-CCP antibodies [55,56]. Tobacco consumption has been associated with epigenetic modifications, particularly methylation in the *HLA* region, which is higher in smokers with positive anti-CCP who carry the *HLA-DRB1* allele. The association between tobacco consumption and RA remains a subject of controversy [36]. Other variables such as diet, physical activity, and alcohol consumption were not considered in our study. It is worth noting that consuming fish three times a week, as well as moderate alcohol consumption and tea, has been associated with a reduced risk or protective effect. Surprisingly, a study in Northern Sweden showed that salt intake increases the risk of RA by 2.26 times in smokers but not in non-smokers. Meanwhile, coffee consumption was associated with a higher occurrence of RA [45,57].

Regarding commonly used biomarkers in the clinical evaluation of RA, we observed statistically significant values for CRP, RF, and anti-CCP among patients with RA and healthy individuals serving as the control group in each of the populations. However, we highlight that higher CRP values in the control group were found when compared to RA patients, which is in contrast with the RA cases from the Western population included in this study and other previously studied populations. The higher CRP levels could be an indicator of metabolic disequilibrium in any inflammatory process [58,59,60]. 

Conversely, the values obtained for anti-CCP in the Western group of patients with RA exceeded those observed in cases from the South; Duran Avelar and colleagues documented similar values in Nayarit, Mexico, as those found in Jalisco (Western Mexico) [45]. Nevertheless, our findings indicate that patients originating from Guerrero (Southern Mexico) exhibited elevated RF levels in comparison to individuals from Jalisco. The values align with those documented in a study conducted in Singapore [46].

Association analyses between the *rs7574865* variant and RA revealed significant values in the dominant additive model and the associated genotypes, as well as the risk allele, but not in the recessive model. We found that the OR obtained for the risk allele T in the Jalisco population (OR = 1.915) was similar to previous reports by other groups in Mexico, Italy, Japan, Spain, Colombia, Egypt, China, and Slovakia [61,62,63,64]. A proportion of 73.3% of the RA patients carried genotypes with the *STAT4* T allele (GT + TT); within this group, 50.8% were heterozygous for the variant allele (GT) and 18.3% were homozygous (TT), which is similar to a previous report in the Mexican population [61]. On the other hand, this variant (*rs7574865)* showed no association in the Southern population. In the case of the *rs897200* variant, it was not associated with the disease or disease activity in any studied populations. Currently, there is no evidence of the association with RA or different immunologic disorders, but this variant is associated with BD [31,65,66].

Our results indicate that the T allele of the *rs7574865* variant of the *STAT4* gene is also associated with the presence of positive anti-CCP antibodies, moderate-to-high disease levels, and a predisposition to the development of RA in the Western population. However, none of the genotypes are linked to disease activity in the Southern population. These findings align with findings from prior studies conducted in a Mexican population, where the T allele of the *rs7574865* variant was associated with moderate-to-high disease activity as assessed by the DAS28 [61], while in an Egyptian population, the risk allele is associated with RF levels (+) and both positive and negative values of anti-CCP [67]. 

Furthermore, although the two variants studied here were not in linkage disequilibrium, an association study conducted in patients with pulmonary tuberculosis discovered an association between the *rs897200* variant and the *rs7572482* and *rs1031509* variants of the *STAT4* gene [32,68]. A study conducted in China that aimed to investigate BD revealed that the three identified risk single nucleotide variants (SNVs), *rs897200, rs7574070*, and *rs7572482,* were located within the same linkage disequilibrium block. However, the *rs7574865* variant in the *STAT4* gene was found to be in a different linkage disequilibrium block compared to the SNVs *rs897200, rs7574070*, and *rs7572482*, and it did not show any association with BD. These results imply a robust association among the three variants and BD. Additionally, previous studies have demonstrated that the SNV *rs7574865* in *STAT4* is associated with several autoimmune diseases. This evidence proposes that *STAT4* may function as a common risk factor in various autoimmune diseases, indicating that the block associated with BD might be distinct from the block associated with other autoimmune diseases such as RA and SLE [31,46,65].

Regarding the functionality of the studied variants and potential relevance to RA, for *rs897200*, the presence of the T allele leads to the binding of multiple transcription factors to DNA, enhancing mRNA expression. Conversely, the C allele shows no such binding, resulting in reduced mRNA expression. This variant is implicated in regulating genes involved in inflammation, although no direct association with RA has been established [31,32,65]. On the other hand, the T allele of the *rs7574865* variant is associated with increased *STAT4* mRNA and protein levels in RA patients [30]. The functional significance of this variant is complex due to its location in the 3rd intron of the gene, a region not typically known for encoding proteins. However, the concept of Intron-Mediated Enhancement (IME) suggests that introns can play a significant role in gene expression regulation. This idea is supported by in silico studies that locate *rs7574865* near distal enhancers and important transcription factors like CTCF, highlighting its potential regulatory function in gene expression [69,70,71,72].

Finally, it is important to look into how the JAK/STAT signaling pathway affects the disease’s development, since these variants may change the expression of mRNA and proteins. While this study showed a significant finding for RA, it is important to exercise caution due to some limitations of this study, such as the study design and sample size, which may have an impact on the obtained results. In addition, it is imperative to take into account lifestyle variables, such as the nutritional components relevant to individuals with RA, in order to explore a potential correlation between dietary parameters, including the consumption of specific foods and their frequency, in forthcoming research endeavors.

## 5. Conclusions

In this study, the T allele of the *rs7574865* variant is considered a risk allele for the susceptibility to develop RA. Its presence is also associated with medium–high disease activity and positive anti-CCP values in the population of Jalisco. On the other hand, no significant values were found for the *rs897200* variant in either of the two studied populations.

## Figures and Tables

**Table 1:** Comparison of clinical and sociodemographic variables between groups for each population.

Variable	Southern Controls (*n* = 120)	Southern Cases (*n* = 120)	* *p*	Western Controls (*n* = 116)	Western Cases (*n* = 120)	* *p*
**Demographics**						
Age (years) ^f^	46 (35–56)	44 (36–58)	0.770 ^b^	38 (30–50)	51 (42–61)	<0.001 **^b^
**Gender**						
Female/Male ^g^	92.5 (111)/7.5 (9)	91.7 (110)/8.3 (10)	0.811 ^c^	89.7 (104)/13.3 (16)	82.8 (96)/17.2 (20)	0.404 ^c^
Family history of RA ^g^	-	25 (30)	-	-	60.8 (73)	<0.001 **^c^
**Clinical assessment**						
Time of disease evolution (years) ^d,f^	-	6 (3–11.75)	-	-	6 (2–14)	0.930 ^b^
DAS28 ^d,g^						
Remission <2.6 ^g^	-	17.5 (21)	-	-	12.5 (15)	<0.001 **^c^
Low activity ≥2.6 to < 3.2 ^g^	-	41.7 (50)	-	-	10.8 (13)	<0.001 **^c^
Moderate activity ≥3.2 to <5.1 ^g^	-	28.3 (34)	-	-	47.5 (57)	<0.001 **^c^
High activity >5.1 ^g^	-	12.5 (15)	-	-	29.2 (35)	<0.001 **^c^
HAQ (0–3) ^d,f^	-	0.2050 (0.04–0.6050)	-	-	1.1150 (0.3725–1.87)	<0.001 **^b^
VAS ^d,f^	-	40 (20–50)	-	-	40 (20–50)	0.419 ^b^
ESR (mm/h) ^d,e^	-	32.18 ± 14.374	-	-	36.61 ± 17.082	0.030 *^a^
CRP (mg/L) ^f^	26.50 (3–58)	15.75 (2.3–298.2)	**0.002 ^b^**	0.2650 (0.0950–0.9485)	1.53 (0.34–5.68)	<0.001 **^b^
RF (IU/mL) ^f^	11.75 (0–326)	181.45 (0–300)	<0.001 **^b^	8 (6.30–9.48)	101.10 (43.8–288.9)	<0.001 **^b^
Anti-CCP (U/mL) ^e^	0.00 (0–100)	148.42 (0–900)	<0.001 **^b^	8.04 ± 8.736	253.67 ± 179.21	<0.001 **^a^
**Treatment**						
Corticosteroids ^d,g^	-	42.5 (51)	-	-	13.3 (16)	<0.001 **^c^
DMARDs ^d,g^						
Methotrexate ^d,g^	-	81.7 (98)	-	-	88.3 (106)	<0.001 **^c^
Chloroquine ^d,g^	-	42.5 (51)	-	-	13.3 (16)	<0.001 **^c^
Sulfasalazine ^d,g^	-	24.2 (29)	-	-	72.5 (87)	<0.001 **^c^

Anti-CCP = Anti-cyclic citrullinated peptide antibodies; CRP = C-reactive protein; DAS28 = index of disease activity; ESR = erythrocyte sedimentation rate; EVA PAC = pain scale; HAQ = Health assessment questionnaire; RF = rheumatoid factor; VAS = pain measurement with visual analog scale. ** significance value < 0.001; * significance value < 0.05; ^a^ Student’s *t*-test; ^b^ Mann–Whitney U-test; ^c^ Chi-square test; ^d^ comparison between cases of both populations; ^e^ mean and standard deviation; ^f^ median and ranges; ^g^ frequencies and percentages.

**Table 2:** Association of *rs7574865* and *rs897200* genotype variants of *STAT4* gene in the population of Western and Southern Mexico with diagnostic variables.

Southern (Control/Cases)							
Clinical Assessment	Genotypes			OR (95% CI)		OR (95% CI)	
*STAT4*: *rs7574865* T > G	GG	GT	TT	GT	*p*	TT	*p*
DAS28 (<3.20)	44/18	56/30	20/12	1.310 (0.647–2.651)	0.453	1.467 (0.595–3.613)	0.700
DAS28 (>3.20)	44/14	56/30	20/15	1.684 (0.798–3.554)	0.169	2.357 (0.958–5.797)	0.059
RF-negative (<20 IU/mL)	32/4	47/7	16/3	1.191 (0.322–4.407)	0.792	1.50 (0.299–7.525)	0.620
RF-positive (>20 IU/mL)	12/28	9/53	4/24	2.524 (0.949–6.712)	0.059	2.571 (0.732–9.03)	0.132
Anti-CCP-negative (<5 U/mL)	39/3	52/5	18/2	1.250 (0.282–5.548)	0.768	1.444 (0.222–9.413)	0.699
Anti-CCP-positive (>5 U/mL)	5/29	4/55	2/25	2.371 (0.591–9.514)	0.213	2.155 (0.384–12.094)	0.374
***STAT4: rs897200* T > C**	**CC**	**CT**	**TT**	**CT**	***p***	**TT**	***p***
DAS28 (<3.20)	69/34	40/22	11/4	1.116 (0.575–2.166)	0.745	0.738 (2.19–2.489)	0.623
DAS28 (>3.20)	69/41	40/16	11/3	0.673 (0.335–1.351)	0.264	0.459 (0.121–1.742)	0.243
RF-negative (<20 IU/mL)	56/6	31/7	8/1	2.108 (0.651–6.827)	0.206	1.167 (0.124–10.99)	0.892
RF-positive (>20 IU/mL)	13/69	9/31	3/6	0.649 (0.251–1.677)	0.37	0.377 (0.83–1.701)	0.190
Anti-CCP-negative (<5 U/mL)	64/7	35/4	10/0	1.045 (0.286–3.818)	0.947	0.410 (0.022–7.716)	0.298
Anti-CCP-positive (>5 U/mL)	5/68	5/34	1/7	0.50 (0.135–1.846)	0.291	0.515 (0.052–5.050)	0.562
**Western** **(control/cases)**							
**Clinical Assessment**	**Genotypes**			**OR (95% CI)**		**OR (95% CI)**	
***STAT4*: *rs7574865* T > G**	**GG**	**GT**	**TT**	**GT**	***p***	**TT**	***p***
DAS28 (<3.20)	60/6	45/14	11/1	3.111 (1.109–8.728)	0.025 *	0.909 (0.099–8.307)	0.932
DAS28 (>3.20)	60/22	45/40	11/16	2.424 (1.268–4.635)	0.006 *	3.967 (1.597–9.854)	0.002 *
RF-negative (<20 IU/mL)	37/4	27/8	8/3	2.741 (0.748–10.044)	0.118	3.469 (0.646–18.625)	0.130
RF-positive (>20 IU/mL)	2/22	0/46	0/14	10.333 (0.476–224.342)	0.046	3.222 (0.144–72.049)	0.267
Anti-CCP-negative (<5 U/mL)	23/2	19/0	3/0	0.241 (0.011–5.324)	0.206	1.343 (0.053–34.198)	0.611
Anti-CCP-positive (>5 U/mL)	37/26	26/54	8/17	2.956 (1.489–5.869)	0.001 **	3.024 (1.137–8.046)	0.023 *
***STAT4: rs897200* T > C**	**CC**	**CT**	**TT**	**CT**	***p***	**TT**	***p***
DAS28 (<3.20)	48/14	51/6	17/5	0.403 (0.143–1.135)	0.078	1.008 (0.316–3.221)	0.988
DAS28 (>3.20)	48/42	51/40	17/7	0.896 (0.499–1.610)	0.714	0.471 (0.178–1.245)	0.123
RF-negative (<20 IU/mL)	24/7	37/5	11/3	0.463 (0.132–1.629)	0.223	0.935 (0.203–4.315)	0.931
RF-positive (>20 IU/mL)	0/46	2/41	0/9	0.178 (0.008–3.826)	0.139	0.204 (0.004–10.951)	1.000
Anti-CCP-negative (<5 U/mL)	25/3	13/0	7/0	0.270 (0.013–5.617)	0.220	0.486 (0.022–10.495)	0.365
Anti-CCP-positive (>5 U/mL)	23/53	38/46	10/12	0.525 (0.274–1.008)	0.051	0.521 (0.197–1.376)	0.184

Anti-CCP = anti-cyclic citrullinated peptide antibodies; DAS28 = score of disease activity; RF = rheumatoid factor; RA = Rheumatoid Arthritis; OR = odds ratio; CI = Confidence interval, genotype GG is the reference. ** significance value <0.001; * significance value < 0.05.

**Table 3:** Association of genotypes of *rs7574865* and *rs897200* variants of *STAT4* gene in the population of Western and Southern Mexico with RA.

	Southern				
Genotype	RA (*n* = 120) % (*n*)	Controls (*n* = 120) % (*n*)	OR	95% IC	*p*-Value
*STAT4: rs7574865 T > G*					
GG ^1^	26.7 (32)	36.7 (44)	Reference		
GT ^2^	50.8 (61)	46.7 (56)	1.498	(0.837–2.681)	0.172
TT ^3^	22.5 (27)	16.7 (20)	1.856	(0.889–3.875)	0.097
GT + TT ^4^	73.3 (88)	63.4 (76)	1.592	(0.919–2.757)	0.095
GG + GT ^5^	77.5 (93)	83.4 (100)	Reference		
TT ^3^	22.5 (27)	16.7 (20)	1.452	(0.763–2.763)	0.254
G ^6^	52.1 (125)	60.05 (144)	Reference		
T ^7^	47.9 (115)	40.05 (96)	1.380	(0.961–1.981)	0.080
*STAT4: rs897200 T > C*					
CC ^1^	62.5 (75)	57.5 (69)	Reference		
CT ^2^	31.7 (38)	33.3 (40)	0.874	(0.504–1.517)	0.632
TT ^3^	5.8 (7)	9.2 (11)	0.585	(0.215–1.595)	0.291
CT + TT ^4^	37.5 (45)	42.5 (51)	0.812	(0.484–1.362)	0.429
CC + CT ^5^	94.2 (113)	90.8 (109)	Reference		
TT ^3^	5.8 (7)	9.2 (11)	0.614	(0.230–1.641)	0.326
C ^6^	78.3 (188)	74.1 (178)	Reference		
T ^7^	21.6 (26)	25.9 (62)	0.794	(0.521–1.211)	0.283
**Western**					
**Genotype**	**RA (*n* = 120)** **%(*n*)**	**Controls (*n* = 116)** **%(*n*)**	**OR**	**95% IC**	***p*-Value**
*STAT4: rs7574865 T > G*					
GG ^1^	30.8 (37)	51.7 (60)	Reference		
GT ^2^	50.8 (61)	38.8 (45)	2.198	(1.253–3.857)	0.005 *
TT ^3^	18.3 (22)	9.5 (11)	3.243	(1.412–7.450)	0.004 *
GT + TT ^4^	69.1 (83)	48.3 (56)	2.403	(1.412–4.090)	0.001 *
GG + GT ^5^	81.6 (98)	90.5 (105)	Reference		
TT ^3^	18.3 (22)	9.5 (11)	2.143	(0.988–4.648)	0.05
G ^6^	56.2 (135)	71.1 (165)	Reference		
T ^7^	43.7 (105)	28.9 (67)	1.915	(1.308–2.806)	0.005 *
*STAT4: rs897200 T > C*					
CC ^1^	47.5 (57)	40.5 (47)	Reference		
CT ^2^	41.7 (50)	44.8 (52)	0.793	(0.459–1.371)	0.405
TT ^3^	10.8 (13)	14.7 (17)	0.631	(0.278–1.430)	0.267
CT + TT ^4^	52.5 (63)	59.5 (69)	0.753	(0.450–1.261)	0.280
CC + CT ^5^	89.2 (107)	85.3 (99)	Reference		
TT ^3^	10.8 (13)	14.7 (17)	0.708	(0.327–1.531)	0.378
C ^6^	68.3 (164)	62.9 (146)	Reference		
T ^7^	31.7 (76)	37.1 (86)	0.787	(0.538–1.151)	0.216

* significance value < 0.05; RA = Rheumatoid Arthritis; OR = odds ratio; CI = Confidence interval; ^1^ Wild homozygous genotype *rs7574865* T > G (GG) and *rs897200* T > C (CC); ^2^ Heterozygote genotype *rs7574865* T > G (TT) and *rs897200* T > C (TT); ^3^ Mutated homozygous genotype *rs7574865* T > G (TT) and *rs897200* T > C (TT); ^4^ dominant models *rs7574865* T > G (GT + TT) and *rs897200* T > C (CT + TT); ^5^ recessive models *rs7574865* T > G (GG + GT) and *rs897200* T > C (CC + CT); ^6^ Variant allele *rs7574865* T > G (G) and *rs897200* T > C (C); ^7^ Ancestral allele *rs7574865* T > G (T) and *rs897200* T > C (T).

## Data Availability

The data presented in this study are available upon request to the corresponding author. The data are not publicly available due to the protection of participants’ personal data.
